# TLX activates MMP-2, promotes self-renewal of tumor spheres in neuroblastoma and correlates with poor patient survival

**DOI:** 10.1038/cddis.2014.449

**Published:** 2014-10-30

**Authors:** P L Chavali, R K R Saini, Q Zhai, D Vizlin-Hodzic, S Venkatabalasubramanian, A Hayashi, E Johansson, Z-j Zeng, S Mohlin, S Påhlman, L Hansford, D R Kaplan, K Funa

**Affiliations:** 1 Sahlgrenska Cancer Center at the Sahlgrenska Academy, University of Gothenburg, Box 425, Gothenburg SE 40530, Sweden; 2 Department of Oncology, University of Cambridge, Li Ka Shing Centre, Robinson Way, Cambridge CB2 0RE, UK; 3 School of Chemical and Biotechnology, SASTRA University, Thanjavur 613401, India; 4 Molecular Biology Research Center, School of Biological Science and Technology, Central South University, Changsha, China; 5 Center for Molecular Pathology, Lund University, Skåne University Hospital, Malmö SE 20502, Sweden; 6 Program in Cell Biology, Hospital for Sick Children, Toronto, Canada M5G 1X8; 7 Department of Molecular Genetics, University of Toronto, Toronto, Canada M5S 1A8

## Abstract

Nuclear orphan receptor TLX (*Drosophila* *tailless* homolog) is essential for the maintenance of neural stem/progenitor cell self-renewal, but its role in neuroblastoma (NB) is not well understood. Here, we show that TLX is essential for the formation of tumor spheres in three different NB cell lines, when grown in neural stem cell media. We demonstrate that the knock down of TLX in IMR-32 cells diminishes its tumor sphere-forming capacity. In tumor spheres, TLX is coexpressed with the neural progenitor markers Nestin, CD133 and Oct-4. In addition, TLX is coexpressed with the migratory neural progenitor markers CD15 and matrix metalloproteinase-2 (MMP-2) in xenografts of primary NB cells from patients. Subsequently, we show the effect of TLX on the proliferative, invasive and migratory properties of IMR-32 cells. We attribute this to the recruitment of TLX to both MMP-2 and Oct-4 gene promoters, which resulted in the respective gene activation. In support of our findings, we found that TLX expression was high in NB patient tissues when compared with normal peripheral nervous system tissues. Further, the *Kaplan*–*Meier* estimator indicated a negative correlation between TLX expression and survival in 88 NB patients. Therefore, our results point at TLX being a crucial player in progression of NB, by promoting self-renewal of NB tumor-initiating cells and altering their migratory and invasive properties.

Neuroblastoma (NB) is the most common extracranial solid tumor found in children, accounting for 8–10% of childhood cancers that likely originates from neural crest-derived sympathoadrenal progenitor cells. NB cells can express a number of neural stem cell and progenitor markers, including CD133, ABCG2 (ATP binding cassette-G2) and Nestin.^1, 2, and 3^ As self-renewal and differentiation of neural stem cells is predominantly regulated by a number of stem cell fate determinants such as Notch, Wnt, Hedgehog, PTEN (phosphatase and tensin homolog) and TLX (*Drosophila* *tailless* homolog), also named NR2E1,^4, 5, 6, 7 and 8^ it is possible that deregulation of such genes may be responsible for the regulation of tumorigenesis in neural cancers. TLX, an orphan nuclear receptor, is predominantly expressed in the embryonic and adult forebrain, and is a crucial regulator of neurogenesis by regulating neural stem cell self-renewal and maintenance.^8, 9 and 10^

Recently, we reported that TLX upon hypoxia stimulates neural stem cell renewal by promoting Oct-4 transcription in adult hippocampal progenitors.^11^ However, its role in malignancy in the nervous system is not well understood, even though recent studies suggest a role in the initiation of cancer stem cells of glioma.^12, 12^ NB of high malignancy acquires the ability to degrade components of extracellular matrix to penetrate the basal membrane of blood vessels to metastasize by activating matrix metalloproteinases (MMPs). NB cells might express these proteins as the normal neural stem cells are regulated by the subfamily, MMP-2 and MMP-9, also called gelatinases.^14^ In fact, MMP-2 and MMP-9 have been reported to have an important role in invasion and metastasis of glioma and other cancers.^15, 16 and 17^

In this study, we demonstrate that the depletion of TLX in NB cell lines inhibits their sphere-forming capacity and reduces their invasion and migration. We show that the altered migration is a direct function of MMP-2 regulation. On the other hand, under hypoxic conditions, TLX can activate oct-4 gene, promoting self-renewal of tumor spheres. We then correlate TLX levels with patient survival data, pointing at TLX being a crucial player in NB progression.

## Results

### TLX promotes the proliferation and sphere-forming capacity of NB cells

**Figure 1:**
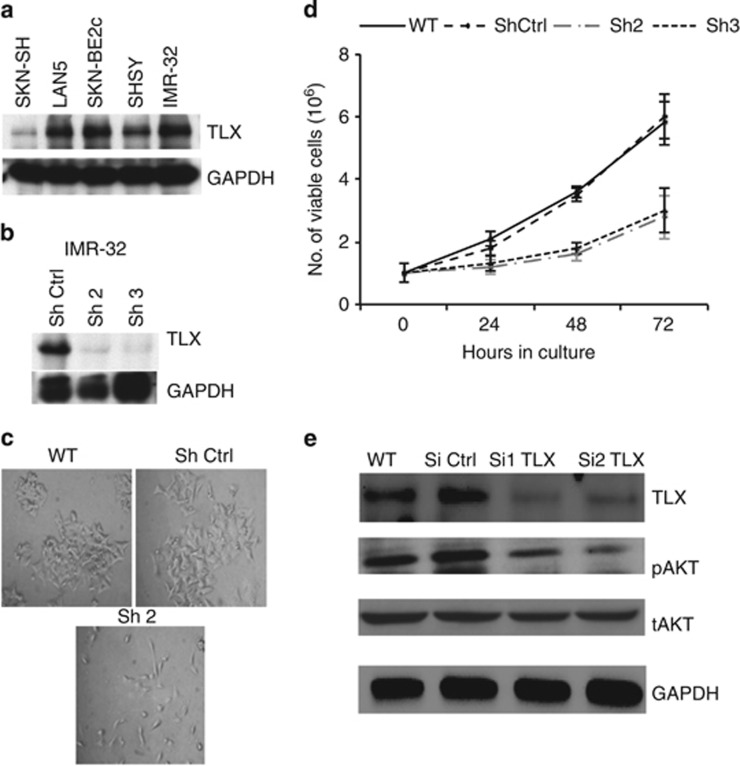
TLX expression is elevated in NB cell lines. (**a**) Immunoblot analysis for TLX in equal amounts of protein from cell extracts of NB cells lines, namely SH-SY5Y, SK-N-SH, SK-N-BE2c, LAN-5 and IMR-32. GAPDH was included as a loading control. (**b**) Immunoblot analysis of TLX in shRNA-control (ShCtrl) or TLX-specific shRNA (Sh2, Sh3)-derived stable clones. (**c**) Phase-contrast image of IMR-32 WT, control and ShTLX cultured under normal proliferation conditions. Magnification, × 20. (**d**) Fold expansion of viable cells at different time points at 24, 48 and 72 h of IMR-32, ShCtrl, Sh2 and Sh3. (**e**) Immunoblot analysis for TLX, P-Akt and total-Akt (T-Akt) in equal amount of proteins from the extracts of IMR-32 WT, Si Ctrl and transiently silenced TLX Si1 and Si2 cell lines. GAPDH was used as a loading control

We first examined the protein levels of TLX in different NB cell lines, including SH-SY5Y, SK-N-SH, SK-N-BE2c, LAN-5 and IMR-32 (Figure 1a). TLX was expressed at higher levels in SK-N-BE2c, IMR-32 and LAN-5 when compared with the other cell lines. For further studies, we used IMR-32 cells where TLX was stably knocked down using shRNAs. As shRNAs 2 and 3 gave 80% reduction in the protein levels (Figure 1b), further experiments were carried out using clones generated from shRNAs 2 and 3. We next validated the growth characteristics and proliferation potential of TLX-silenced clones and compared them with the wild-type (WT) parental IMR-32 cells. Stably silenced clones were prone to detachment after seeding, but surviving cells showed neurite-like processes (Figure 1c). The doubling time of WT and Sh-control IMR-32 cells was ~24 h, whereas those of the TLX-silenced clones were 56–72 h, estimated by MTT cell viability assays (Figure 1d). Interestingly, the relative number of viable cells in each passage in the TLX-depleted cells decreased by twofold as compared with the parental cells (Figure 1d). Our previous study showed the depletion of TLX in adult hippocampal progenitors increased active caspase-3, indicative of a prosurvival role for TLX in neural progenitors.^11^ As Akt is a well-known prosurvival signaling molecule and its activation is a marker for poor outcome and prognosis in NB,^18^ the levels of phosphorylated Akt were compared in WT cells before and after transient knockdown of TLX using shRNA. pAkt was significantly reduced upon transient TLX knockdown (Si1 and Si2) as compared with WT and control SiRNA-transfected IMR-32 cells (Figure 1e). These results suggest that TLX mediates survival by maintaining pAkt levels, perhaps via its role as a PTEN transcriptional repressor.^19^

### TLX is enriched in self-renewing spheres derived from NB cells

**Figure 2:**
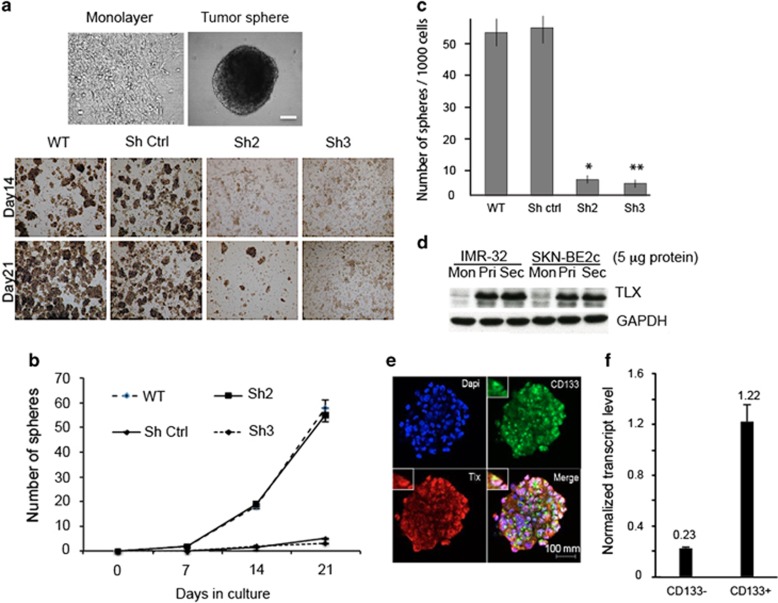
TLX is essential for tumor sphere formation. (**a**) Representative images of monolayer (contains serum) and IMR-32 spheres (serum-free). Bar, 20 *μ*m. Lower panel depicts representative images obtained by sphere formation assays. IMR-32 WT, ShCtrl, Sh2 and Sh3 cells were cultured for 2–3 weeks in the defined media for sphere formation and spheres collected and counted after indicated time intervals. (**b**) Quantitation of the number of spheres after indicated time intervals in control or TLX-silenced cells. (**c**) Number of spheres per 1000 cells derived from primary spheres in subsphere formation assay. (**d**) Immunoblot analysis of monolayer (Mon), primary (Pri) or primary-derived secondary spheres (Sec) of IMR-32 cells for TLX expression. GAPDH is used as loading control. (**e**) Immunofluorescence image of IMR-32 spheroid double stained for CD133 and TLX (bar, 100 *μ*m) and the larger magnification (bar, 20 *μ*m). (**f**) TLX transcript levels were measured by qPCR and normalized to GAPDH in CD133-positive and -negative cells derived from from single-cell suspension of spheroids sorted using CD133 Microbead Kit (Miltenyi Biotec). Control set to 1±S.D.

NB cell lines and NB cells metastasizing to bone marrow have earlier been demonstrated to harbor tumor-initiating cells (TICs), which can then be isolated by growing them in stem cell media.^1, 20^ Considering that TLX is essential for maintenance and self-renewal of neural stem cells, we investigated if TLX could have a similar role in maintaining the population of NB-TICs. For this purpose, 1 × 10^5^ WT or TLX-silenced IMR-32 cell clones were reseeded in serum-free media containing N2 supplement, basic fibroblast growth factor (bFGF) and epidermal growth factor (EGF), and grown for a period of 21 days with a medium change every third day (Figure 2a, top panel). After 7 days, distinct sphere formation was observed in WT and Sh-control cells, but Sh2 and Sh3 clones showed poor sphere formation ability, even after 21 days, suggesting a requirement of TLX for sphere formation (Figures 2a (bottom panel) and b). To evaluate clonogenic potential, spheres from each of the WT and TLX-silenced cells were dissociated and reseeded at a density of 1000 cells per well and analyzed for secondary sphere formation as an indicator of self-renewal potential. We found that while WT or shRNA-control cells formed 50–60 spheres per well, TLX-silenced stable cells formed only 2–5 spheres per well (Figure 2c). A strong evidence for the role of TLX in sphere was demonstrated when we found a three-to fourfold increase in TLX protein expression in the same number of cells in primary and secondary spheres compared with the monolayer cells in both SK-N-BE2c and IMR-32 cells (Figure 2d). Further, upon IF analysis we found that the spheres coexpressed TLX and CD133 (Figure 2e, left panel). We also sorted these spheres into CD133-positive and -negative fractions using CD133 Microbead Kit (Miltenyi Biotec, Bergisch Gladbach, Germany) and isolated RNA from these cells. We found that TLX transcript was enriched by sixfold in CD133-positive cells, when normalized to glyceraldehyde-3-phosphate dehydrogenase (GAPDH) (Figure 2f).

### TLX enrichment in spheres correlates with proliferation and markers of neural stemness

**Figure 3:**
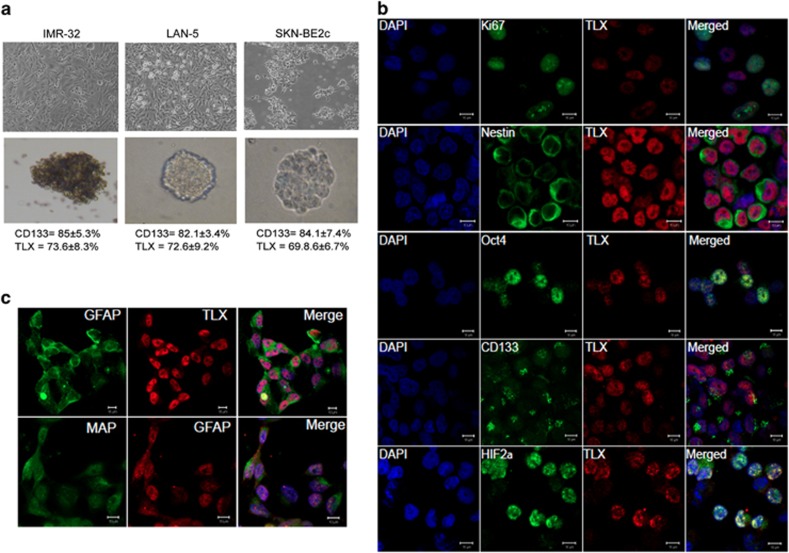
TLX is enriched in proliferative cells of spheres. (**a**) Representative images of monolayers and spheroids from IMR-32, LAN-5 and SK-N-BE2c cells. The numbers below indicate the percentage of TLX- and CD133- expressing cells analyzed by FACS. (**b**) Dispersed SK-N-BE2c spheres stained for TLX (red) and indicated proteins (green) (right panel). Images were obtained by confocal microscopy. Bar, 10 *μ*m. (**c**) Single-cell suspension of spheres were differentiated using 1% FBS and cells were stained for coexpression of TLX using differentiation markers GFAP and MAP2ab

To identify if TLX is coexpressed with CD133 in tumor spheres from different cell lines, we assayed the spheres from LAN-5 and SKN-BE2c cells for coexpression of TLX and CD133 using FACS. Both the cell types expressed similar levels of TLX and CD133, comparable to IMR-32 (Figure 3a). We additionally stained for various proliferation and neural stem cell markers in SK-N-BE-2c cells dispersed from spheres (Figure 3b). We found that these sphere-forming cells showed an overlap of TLX with Ki67, Nestin, Oct-4, CD133 and hypoxia-inducing factor-2*α* (HIF-2*α*). Among them, Nestin and CD133 were detected in the cytoplasm and membrane, respectively. Many TLX-positive cells were proliferating, as shown by nuclear Ki67 staining. Indeed, these spheres could be differentiated by the addition of FBS to express MAP2ab and GFAP (Figure 3c). These results indicate that the tumor spheres have neural stem cell-like characteristics.

### TLX is expressed in xenograft tissues of primary NB-TICs derived from patients

**Figure 4:**
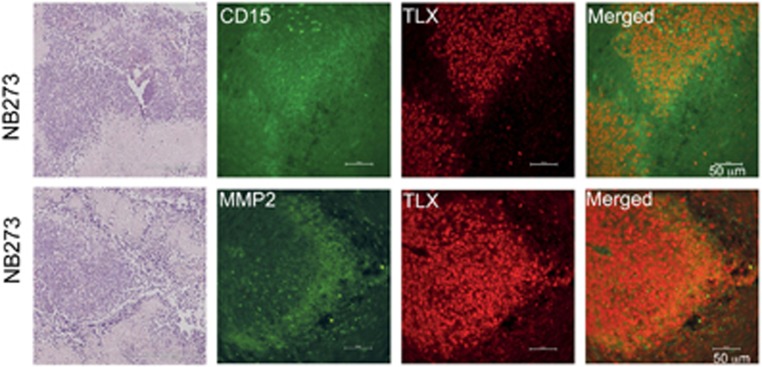
Xenografts of NB-TIC lines express CD15 and MMP-2 in tumor sections overlapping with or adjacent to TLX. Sections from the xenografts were stained with double immunofluorescence for TLX/CD15 or TLX/MMP-2, and representative images are shown. TLX (red) and CD15/MMP-2 (green). Scale bar represents 50 *μ*m. Tissue structure is shown by HE staining. Scale bar represents 11 × 200 *μ*m

To corroborate our findings from cell lines, we examined the coexpression of TLX with neural progenitor marker CD15, which additionally marks migratory neuronal progenitors. For this, we used xenografts from primary human patient-derived NB-TICs (Figure 4). Paraffin-embedded tissue sections from xenografts recovered from non-obese diabetic/severe-combined immunodeficiency (NOD/SCID) mice grafted with NB-TICs were stained with antibodies for TLX and markers for migratory neural progenitors (CD15). In these xenografts, tightly aggregated TLX-positive cells were surrounded by cells expressing low levels of CD15 or CD15-negative areas. Some of these areas were necrotic with many inflammatory cells. Interestingly, most of the TLX-expressing cells also expressed MMP-2 that is secreted in large amounts, likely to facilitate ECM degradation and tumor dissemination, a hallmark of advanced stages of NB. Several xenografts derived from other primary NB cell lines showed a similar pattern of MMP-2 and CD15 staining with the relation to the TLX staining, although intensity of TLX staining varied among the cell lines (not shown).

### TLX increases migratory and invasive properties of NB cells

**Figure 5:**
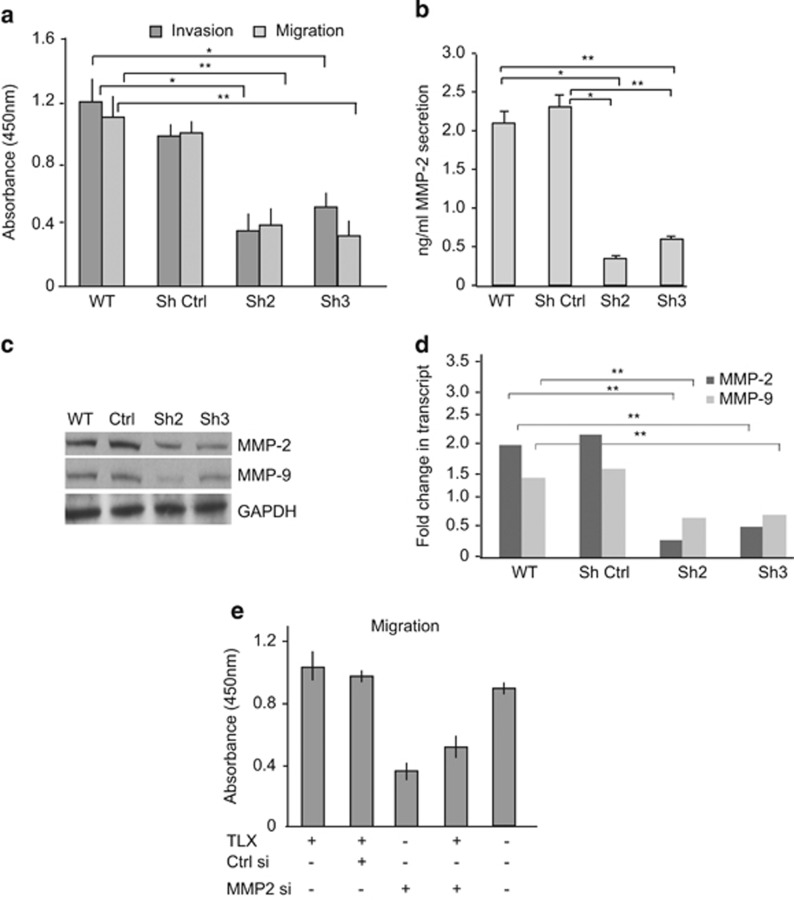
TLX promotes migration and invasion in IMR-32 cells. (**a**) Invasion and migration assays were performed as described in Materials and Methods using WT IMR-32, shRNA-control (ShCtrl) or Sh2 and Sh3 lines. Values depict the absorbance at 450 nm, representing the invasion/migration index values. (**b**) Graph depicting the increase of secreted MMP-2 levels in the conditioned media of WT, ShCtrl, Sh2 and Sh3 cells measured by ELISA. (**c**) Immunoblot analysis of MMP-2 and MMP-9 from conditioned media of control or shTLX cells. Remaining cells in the plate were lysed and used for GAPDH control. (**d**) Fold change in the MMP-2 and MMP-9 transcript, calculated by normalization against GAPDH in WT or TLX-silenced IMR-32 cells. (**e**) Migration assay in IMR-32 cells as described in (**a**), with the indicated transfections below

Staining of NB cell lines and NB-TIC xenograft tissues revealed the co- or juxtalocalization of TLX and MMP-2 and CD15, in particular at the edges of TLX-expressing tumor clusters, suggesting them to be migratory cells. As neural stem cells have a migratory capacity, we asked whether TLX could also promote NB cell migration and invasion. Using a colorimetry-based assay for quantifying migration and invasion separately, we observed that TLX-silenced IMR-32 cells had a two- to threefold reduced migration ability as compared with dispersed sphere-forming WT or control transfected IMR-32 cells (Figure 5a). Similar results were obtained in the invasion assays where the TLX-silenced cells showed a two- to threefold decrease as compared with WT or control cells. We then asked whether the secretion of MMPs known to be involved in migratory and invasive behavior of cancer cells is altered. Using ELISA, we observed a three- to fourfold reduction of secreted MMP-2 in the TLX-silenced cells (Figure 5b). These results were verified by blotting for MMP-2 and MMP-9 levels secreted in the conditioned media from shRNA-control or TLX-silenced cell lines (Figure 5c). This prompted us to investigate the possible role of TLX in gene regulation of MMP-2. To determine if TLX modulates the transcription of MMP-2, we performed RT-PCR analysis of the WT and TLX-silenced clones, and observed a 3.4-fold decrease in MMP-2 transcript levels (Figure 5d). We also observed a more moderate 1.8-fold decrease in MMP-9 mRNA expression. These results suggested the involvement of TLX in activating MMP-2 expression. To rule out a cell line-specific effect of TLX on MMP-2, we validated these results in SKN-BE2c cells. We performed rescue experiments with SKN-BE2c by simultaneous expression of siMMP-2 and TLX by western blot (Supplementary Figure 1).^21^ We observed a 1.8-fold increase in the pro-MMP-2 level upon TLX overexpression, and simultaneous expression of siMMP-2 and TLX rescued the decrease of MMP-2 level by the silencing effect. This is consistent with TLX being an activator of MMP-2 expression. To confirm the MMP-2-mediated promigratory role of TLX, we silenced MMP-2 with siRNA and after 24 h overexpressed TLX in IMR-32 cells. In the absence of MMP-2, TLX overexpression did not result in an significantly increased migratory activity seen with the control cells, indicating the dependence of TLX on MMP-2 for promoting the migration of NB cells (Figure 5e). In summary, TLX alters the migratory capacity of NB cells by inducing MMP-2 levels.

### TLX increases binding to the MMP-2 and Oct-4 promoters in NB cells upon hypoxia

**Figure 6:**
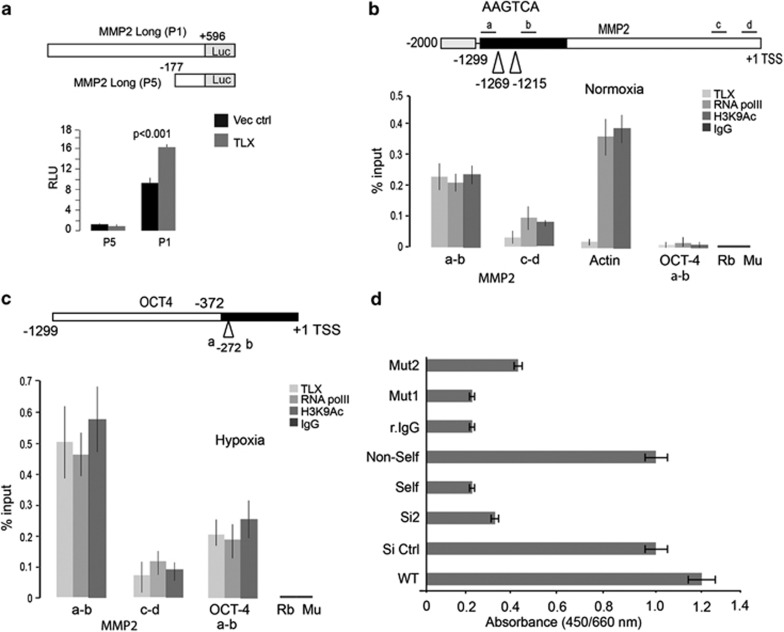
TLX transcriptionally regulates MMP-2 and Oct-4 in hypoxic NB cells. (**a**) Luciferase activity in 293T cells after co-transfection of MMP-2 promoter-luciferase constructs with TLX or control vector. (**b** and **c**) Top panels depict schematic representation of regions analyzed by ChIP within MMP-2 promoter or Oct-4 promoter (**c**). Occupancy of TLX, Pol-II and H3K9 acetylation across the 1.2 kb upstream regulatory regions of TLX-regulated genes *MMP-2* and *OCT-4*, and control actin promoter was monitored by ChIP analysis upon normoxia (**b**) or hypoxia (**c**). Chromatin was isolated from normoxia- or hypoxia-treated cells and ChIP analysis was performed as described in Materials and Methods. Amplicon from each immunoprecipitate is represented as the percentage of input. Each error bar indicates standard deviation calculated from triplicates. (**d**) Graph represents the binding of TLX to MMP-2 promoter as a function of absorbance at 450/650 nm. Biotin-labeled consensus oligos were used to capture TLX of nuclear lysate from WT IMR-32. A nonspecific capture oligo served as control, and rabbit IgG were used to exclude nonspecific binding. Mutant oligos (Mut1 or Mut2) were used to confirm the specificity of capture. The values obtained are means of three independent experiments along with S.D. as error bars

We next examined if TLX regulated the expression of MMP-2 by binding to its promoter. First, we analyzed the effect of TLX overexpression on MMP-2 promoter-luciferase constructs, (a) using a 1780 bp full-length promoter upstream of the transcriptional initiation site and (b) a small promoter construct spanning 177 bp upstream sequence. We identified that while the full-length promoter activity increased by 1.8-fold on TLX overexpression, there was a negligible effect on the shorter construct (Figure 6a).

We then proceeded to analyze the hMMP-2 promoter for putative TLX-binding sites. We identified two ‘AAGTCA' sites binding TLX at ~1.2 kb upstream of the transcription start site (Figure 6b). Quantitative chromatin immunoprecipitation (ChIP) assays by using chromatin isolated from IMR-32 WT cells revealed a basal level binding of TLX to the MMP-2 promoter, along with RNA polymerase-II (Pol-II) recruitment and acetylated H3K9 (H3K9Ac). Under the same conditions, TLX did not bind to *β*-actin promoter. As we have previously shown that TLX is a significant contributor to angiogenesis upon hypoxia, we tested if TLX-mediated MMP-2 regulation is affected upon hypoxia. ChIP of IMR-32 cells when grown in a 1% O_2_ concentration showed that TLX binding to the MMP-2 promoter increased 2–2.5-fold, which correlated with an increased recruitment of Pol-II and H3K9Ac (Figure 6c). In contrast, no occupancy of TLX was detected at the proximal promoter even in hypoxia. The precipitated DNA was sequenced for confirmation (data not shown). A study from our group has identified binding of TLX on the Oct-4 promoter in neural progenitor cells upon hypoxia, leading to self-renewal of these cells and a further activation of Akt signaling pathways.^11^ Recent studies demonstrate the role of Oct-4 in promoting migration and invasion of bladder cancer cells by expression and activation of MMP-2 and MMP-9.^22^ In agreement with this, we found that TLX in IMR-32 cells upon hypoxia was recruited to the human Oct-4 core promoter (Figure 6c). The binding to Oct-4 promoter under normoxic conditions was negligible and comparable to preimmune controls (Figure 6a).

Additionally, we tested TLX-specific binding on the MMP-2 promoter consensus element by performing TLX capture using a biotinylated oligonucleotide encompassing the consensus element of TLX-binding site in the MMP-2 promoter. The biotinylated oligonucleotide was incubated with the nuclear lysate containing TLX, which was captured by a TLX-specific antibody, followed by incubation with a secondary antibody conjugated with horseradish peroxidase (HRP). Color was developed by TMB/E substrate and the binding intensity was calculated using absorption at 450/650 nm. Nonspecificity was ruled out using random IgG, and the non-biotinylated consensus oligonucleotide was used as a competitor to validate the specific binding. Further mutation of the consensus site at the first two bases (Mut1) or the middle three bases (Mut2) markedly reduced the binding of TLX to the probe. Our results show a 4.5-fold enrichment of TLX binding on the MMP-2 promoter site compared with the preimmune control (Figure 6d).

### TLX is expressed in NB tissues derived from patients

**Figure 7:**
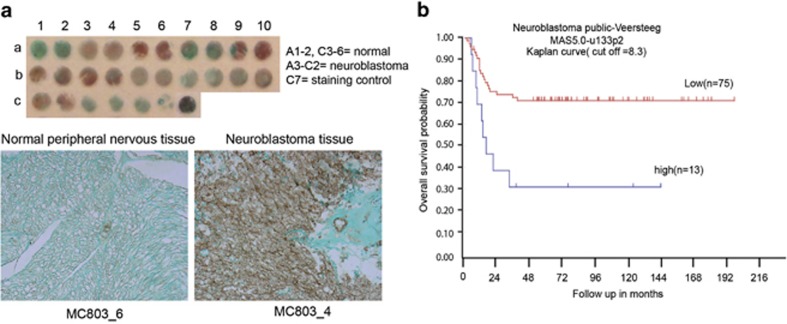
TLX is expressed strongly in NB tissues and correlates with poor survival. (**a**) Low magnification ( × 1) of the whole tissue array stained for TLX. Identity of tissues is described below. Representative photomicrographs of normal peripheral nerve tissue and NB tissue in tissue array are immunostained for TLX (brown) and then counterstained with light green. Magnification, × 40. (**b**) *Kaplan–Meier* analysis of the data from 88 cases of NB, indicating negative correlation of NR2E1 expression with survival

We further examined if we could capture an enrichment of TLX expression in patient samples. For this, we screened NB tumor tissue arrays including 10 human cases (ages 5–48 years, two tissues per case) of aggressive NB and two cases of normal peripheral nervous tissues (PNS) for the expression of TLX (Figure 7a). There was an enhanced TLX expression in these tumors compared with normal PNS tissue. We also used the open R2 statistics application (microarray analysis and visualization platform; http://r2.amc.nl) using microarray data from 88 cases of NB-Versteeg-88 MAS5.0-u133p2 (http://hgserver1.amc.nl/cgi-bin/r2/main.cgi). A *Kaplan–Meier* analysis indicated that the higher expression of TLX (NR2E1) correlates with shorter survival of NB patients, with a cutoff at 8.3, *χ*^2^=9.98, d.f.=1, *P*=0.0016 (Figure 7b).

## Discussion

It has been recognized that a number of stem cell renewal factors are involved in tumorigenesis. TLX is a neural cell-specific renewal factor, and gene amplification of TLX has been reported to occur in malignant glioma.^13^ By expressing TLX, the tumor cells appear to engage neurogenetic niches for their own maintenance.^23^ Here we demonstrate that TLX is also highly expressed in the stem cell-like population enriched from NB, originating from the sympathetic nervous system. Some glioma cells are derived from neural stem cells that are normally maintained in neurogenic niches in the brain.^24^ However, NB is derived from embryonic neural crest cells, arising from the dorsal aspect of neural tube and migrating to the sympathetic ganglia and the adrenal glands. The high expression of TLX observed in the brain of E13.5 mice^25^ indicates the peak of brain neurogenesis. Neural crest cells possess remarkable capacities of migration and multipotency, and begin to migrate around E10.5, detectable in the adrenal glands around E13.5.^26^ The HIF-2*α*-expressing immature neural crest-like NB cells are maintained by perivascular niches^27^ – we have previously showed TLX to stabilize HIF-2*α*.^28^

We have demonstrated that the expression of TLX increases when the NB cells are cultured in neural stem cell media, resulting in tumor sphere formation. Interestingly, these tumor spheres recapitulate neurospheres in their expression of stem cell markers such as CD133, Nestin, Oct-4 and CD15. Furthermore, TLX is expressed in NB-TICs and induces MMPs, which could activate the remodeling of matrix, migration and, possibly, invasion of NB cells. MMP-2 localizes at the migrating edge of TLX-expressing TIC clusters in the xenograft sections of human NB-TICs, suggesting its importance for migratory activities of cancer cells, which may result in invasiveness leading to metastasis. In this context, it is of interest that CD15 in grafted tumor tissues localizes on the surface of TLX-positive cells. CD15, also known as LeX or SSEA-1, is a set of glycan moieties containing fucosylated *N*-acetyllactosamine, which is considered to be important for neural stem cell migration.^29^ In addition, the sialylated or sulfated forms of CD15 is closely associated with lymphocyte rolling, the first step for cellular extravasation, and cancer metastasis.^31, 30^

IMR-32 and NB-TICs express MMP in hypoxia, which might be due to a cooperative effect of TLX and its downstream Wnt signaling. In fact, TLX becomes stabilized in hypoxia,^21^ and has been shown to induce Wnt7b, which subsequently inhibits GSK3.^9^ This leads to stabilization and activation of *β*-catenin, inducing several target molecules such as Myc. We find that TLX expression correlates with pAkt levels,^11^ which could also be a consequence of PTEN repression.^19^ Elevated pAkt can also phosphorylate and inhibit GSK3 apart from stabilizing for HIF-1*α* during hypoxia.^32^ HIF-1*α* also modulates Wnt signaling in hypoxic stem cells and enhances *β*-catenin activation. Thus, we predict that both TLX and HIF might converge and activate signaling pathways through GSK3 inhibition.

While TLX occupies the MMP-2 promoter endogenously, Oct-4 occupancy occurs in a hypoxic milieu, under which conditions these tumor cells would acquire a more epigenetic and phenotypic resemblance to stem cells. Hypoxia is one of the most important contributing factors in the tumor microenvironment, stimulating tumor dedifferentiation and angiogenesis.^33^ In this regard, the expression of HIF-2*α* has been proposed to be associated with dedifferentiation of NB, which may depend on its angiogenic property rather than cell-cycle modulation.^34^ TLX is reported to act as a hypoxia-inducible proangiogenic switch molecule, strongly expressed in postnatal proangiogenic retinal astrocytes, which secrete vascular endothelial growth factor (VEGF) and fibronectin. Moreover, expression of TLX is rapidly downregulated by contact with blood vessels and a derangement of fibronectin matrix was observed in TLX-null mice.^35^ In this context, it is interesting to note that fibronectin fragments from cancer cells can induce the secretion of MMP-2,^36^ whereas MMP-2 and MMP-9 have been shown to degrade fibronectin, as the first step of ovarian cancer metastases.^37^ Thus, TLX affects not only immediate hypoxia-responsive proteins, that is, HIF-2*α* and VEGF, but also affects extracellular matrix proteins needed for vascular organization.

Hypoxia is a well-known condition that induces epithelial-to-mesenchymal transition (EMT), a hallmark of the morphologic changes of tumor cells leading to metastases by various mechanisms.^38^ Interestingly, it has recently been proposed that Oct-4 expression can promote the migration and invasion of glioblastoma cells.^39^ It is an obvious possibility that TLX could be a critical factor by virtue of its dual role in matrix remodeling and angiogenesis, along with regulation of cell-cycle programs contributing to EMT. Further studies are required to show if TLX is a true contributing factor for cancer metastasis by using NB animal models, and whether the metastatic capacity can be altered by depletion of TLX. In sum, our studies propose that TLX employs multiple pathways, amplifying each other to dedifferentiate NB cells and to maintain the progenitor population in a hypoxic environment. The fact that TLX was identified as one of the significant mRNA responders to EGFR network perturbation when analyzed for a prognostic outcome prediction in glioblastoma multiforme,^40^ along with our results, highlight TLX as a crucial candidate for directed cancer therapy.

## Materials and Methods

### Cell culture, transfections and chemical reagents

Non-*MYCN-*amplified cell lines (SK-N-SH and SH-SY5Y) and *MYCN*-amplified cell lines (IMR-32, SK-N-BE2c and LAN-5; ATCC, Manassas, VA, USA) were maintained as described previously.^40^ For tumor sphere formation, cells were cultured in Dulbecco's modified Eagle's medium/Ham's F-12 (1 : 1; Lonza, Basel, Switzerland) containing 1% N_2_ supplement (Invitrogen, Carlsbad, CA, USA), 2% B27 supplement (Invitrogen), 20 ng/ml EGF (Invitrogen), 20 ng/ml bFGF (Invitrogen), 1% L-glutamine (Cambrex, East Rutherford, NJ, USA) and 1% penicillin. Subsphere formation assays were performed by dissociating the primary spheres and seeding them at a density of 1000 cells/well. Differentiation assays were performed by seeding single-cell suspension of spheres into chamber slides (Nalge Nunc Int., Penfield, NY, USA) and culturing them with DMEM/F-12 supplemented with 1% FBS. For silencing TLX, SureSilence shRNA vectors (sequences: Sh1, 5′-TTGCCAGTTTACGTTCTATT-3′ Sh2, 5′-CCGGTTAGATGCTACTGAATT-3′ Sh3, 5′-GCCATTGCAGCCCTTCAAG-AT-3′ Sh4, 5′-CAAGAGGTGGTGGCTCGATTT-3′) were microporated (Digital Bio, Seoul, Korea) into IMR-32 cells. Stable clones were obtained by selection with 40 *μ*g/ml G-418 as described in Seiki.^21^ Transient transfections to silence TLX was carried out with siRNA and appropriate negative control from Superarray Biosciences (Hilden, Germany), using FuGENE HD (Roche, Stockholm, Sweden) according to the manufacturer's protocol.

### MTT assay

Cells were seeded in 96-well plates at 1 × 10^5^ cells per well and the proliferation was measured by the addition of 20 *μ*l 5 mg/ml MTT (3-(4,5-dimethylthiazol-2-yl)-2,5-diphenyl-tetrazolium bromide). After 4 h at 37 °C, medium was removed and formazan crystals were dissolved in DMSO. Absorbance was measured at 570 nm.

### Semiquantitative PCR

Total RNA extraction and cDNA synthesis were carried out according to methods described previously.^11^ PCR was carried out using standard protocol with DreamTaq polymerase (Fermentas, Vilnius, Lithuania). The samples were run in 1.5% agarose gel containing ethidium bromide and analyzed by using FLA 2000 plate reader (Fujifilm, Stockholm, Sweden). Primer sequences are listed below: TLX (62 °C) – sense, 5′-GGCCCATTGTGTATTCCTA-3′ and antisense, 5′-TGAATGGGACCCCAATGTAT-3′ Oct-4 (68 °C) – sense, 5′-ATGGCGGGACACCTGGCTTC-3′ and antisense, 5′-GATTCCTGGCCCTCCAGGAG-3′ actin (62 °C) – sense, 5′-AAGATGACCCAGATCATGTTTGAG-3′ and antisense, 5′-AGGAGGAGCAATGATCTGATCTT-3′ GAPDH (62 °C) – sense, 5′-GAAGGTGAAGGTCGGAGTC-3′ and antisense, 5′-GAAGATGGTGATGGGATTTC-3′ MMP-2 (55 °C) – sense, 5′-TCTCCTGACATTGACCTTGGC-3′ and antisense, 5′-CAAGGTGCTGGCTGAGTAGATC-3′ MMP-9 (52 °C) – sense, 5′-TTGACAGCGACAAGAAGTGG-3′ and antisense, 5′-GCCATTCACGTCGTCCTTAT-3′.

### Immunofluorescence and immunohistochemistry analyses

Cells were cultured on chamber slides and fixed for 20 min with 4% paraformaldehyde in phosphate-buffered saline (PBS). Immunofluorescence staining was performed as described previously.^21^ All primary antibodies were used at a dilution of 1 : 200. Antibodies against TLX (R&D, Abingdon, UK; PP-H6506-00; LifeSpan Biotechnologies, Seattle, WA, USA; LS-B4564), GFAP (Dako, Glostrup, Denmark; Z033429-2), Nestin (Abcam, Cambridge, UK; ab22035), MAP2ab (Sigma-Aldrich, Stockholm, Sweden; M2320), CD133 (Miltenyi Biotech; 130-080-801). Ki67 (SC23900), Oct-4 (SC8630), MMP-2 (SC53630) and HIF-2*α* (SC46691) were also used. For secondary antibodies, IgG conjugated with Alexa Fluor 488/594 (Molecular Probes, Eugene, OR, USA) were used. After nuclear staining with 1 *μ*M Hoechst 33258, slides were mounted with Dako fluorescent medium (Dako), and images were obtained by using confocal microscopy (Zeiss, Oberkochen, Germany).

The deparaffinized formalin-fixed NB-TIC xenograft tissue sections were blocked in 10% NDS/1% bovine serum albumin (BSA)/TBS for 2 h after antigen retrieval in Tris/1 mM EDTA/0.05% Tween-20 solution (pH 9). Primary antibodies used were anti-CD133 (1 : 50; Miltenyi Biotec; 130-090-422), anti-HIF-2*α* (1 : 100; Abcam; ab8365), anti-Nestin (1 : 200; Abcam; ab22035), anti-CD15 (1 : 50; SCB, Dallas, TX, USA; SC19595), anti-MMP-2 (1 : 100; SCB; SC53630) and anti-TLX (LifeSpan Biotechnologies; 1 : 500; LS-B3505). They were diluted in 1% NDS/1% BSA/TBS and applied on tissue sections for overnight incubation at 4 °C. Secondary Alexa Fluor 555/488-conjugated donkey anti-rabbit/mouse IgG (1 : 1000; Invitrogen) were diluted in 1% NDS/1% BSA/TBS and applied on tissues for 1 h. Tissues were counterstained with DAPI (not shown) or hematoxylin and eosin (H&E). H&E-stained slides were analyzed using Leica SCN400 scanner (Leica Microsystems, Wetzlar, Germany) ( × 20). The slides were mounted and analyzed on Nikon Eclipse Ni-E automated upright microscope equipped with Orca Flash 4.0 camera (Hamamatsu, Hamamatsu, Japan), using CFI S Fluor × 40/1.30 Oil (Nikon, Tokyo, Japan) objective. For chromogen staining of deparaffinized tumor tissue arrays (Biomax, Planegg, Germany; MC803), antigen retrieval in 0.01 M citrate buffer (pH 7.2) was carried out, followed by endogenous peroxidase blocking. Sections were then incubated overnight at 4 °C with TLX antibody (1 : 200; R&D; PP-H6506-00). Sections were incubated with HRP-conjugated secondary antibodies at 1 : 1000 dilution (Vector Lab, Burlingame, CA, USA), rinsed and developed by Vector-Red, counterstained with light green and then mounted. Images were recorded by a High Content Microscope (Olympus, Tokyo, Japan). Omission of primary antibodies was used as negative control for staining in immunofluorescence and immunohistochemistry analyses.

### Immunoblot analysis

Cells were cultured on 24-well plates and harvested, and protein was separated on SDS gel, electroblotted onto a PVDF membrane, incubated with 5% BSA in TBS with 0.1% Tween-20 and blotted as described previously.^11^ Antibodies directed against TLX (LifeSpan Biosciences; LS-B4564), pAkt (Cell Signaling, Danvers, MA, USA; 9271) and total-Akt (Santa Cruz Biotechnology, Dallas, TX, USA; SC5298) were used at 1 : 1000; GAPDH (Sigma, St. Louis, MO, USA; G8795) at 1 : 2000; tubulin (Sigma; T8328) and HRP-conjugated anti-mouse or anti-rabbit IgG was used as secondary antibody at 1 : 15 000 (Amersham Biosciences, Buckinghamshire, UK). Signals were detected by enhanced chemiluminescence.

### Animal xenograft

Primary NB cell line (NB273) was cultured as outlined by Hansford *et al.*^20^ Tumors were established using a heterotopic model of tumorigenesis. Briefly, primary NB cells were resuspended in PBS, mixed 1 : 3 with basement membrane extract (Trevigen, Gaithersburg, MD, USA) immediately before injection and injected in a 100 *μ*l volume into the inguinal fat pads of 4- to 5-week-old NOD/SCID mice as approved by University Health Network's Animal Ethics Committee (protocols 09-004 and 2178.5). Animals were monitored weekly for evidence of tumor formation and were killed when tumors reached 1.0–1.5 cm^3^. Tumors were recovered and fixed in 10% formalin for 24 h before paraffin embedding. Tumors were characterized as NB tumors by H&E staining and immunohistochemistry for the NB markers MAP2 (1 : 5000; Sigma) and NB84 (1 : 50; Novocastra, Nussloch, Germany), and determined to be free of Epstein–Barr virus contamination by EBER1 *in situ* hybridization (data not shown).

### Invasion and migration assays

Invasion and migration assays were performed using CytoSelectTM 24-Well (Cell Biolabs Inc.; CBA-100-C) according to the manufacturer's instructions. Briefly, equal numbers of cells in suspension (1 × 10^5^) in DMEM complemented with 2% fetal calf serum (FCS) were added to the upper compartment of the chamber, the bottom of which is a polycarbonate membrane insert with 8- mm pore size in a 24-well plate (insert coated with extracellular matrix membrane for invasion assay and left uncoated for migration assay to use serum as chemoattractant), and kept at 37 °C for 16 h. The lower compartment contained DMEM with 10% FCS. At the end of the incubation period, noninvasive or non-migratory cells from the upper surface of the filter were wiped off with a swab. The lower surface of the filter was stained with a dye and extracted. A volume of 100 *μ*l of each sample was transferred to a 96-well plate and the OD at 560 nm was measured. To detect the amount of MMP-2 secreted by control or by TLX knockdown-stable cells, cells were maintained in serum-free media for 30 h at 37 °C and conditioned media were collected. Conditioned media were diluted 15-fold in diluent buffer from the kit (Invitrogen; KHC3082), added to wells and processed for ELISA along with serially diluted standards and diluent only as negative control. Samples were processed according to the manufacturer's instructions and the results recorded by measuring absorbance at 450 nm. Following collection of conditioned media, cells from different wells were trypsinized and counted to normalize the MMP-2 secreted. Transfection of siMMP-2 (SC29398) was performed as described above. All experiments have been performed as three biological replicates and two technical duplicates. Standard deviation from the mean has been plotted in the graphs.

### MMP-2 promoter-luciferase assay

The MMP-2 promoter-luciferase reporter constructs^23^ were co-transfected with vectors expressing indicated amounts of Flag-TLX or Flag vector alone, 48 h before being lysed and processed using the Luciferase Reporter Assay System (Promega, Madison, WI, USA) according to the manufacturer's instruction.

### ChIP and colorimetric biotin-oligonucleotide transcription factor-binding assay

For the ChIP assay, 1–5 × 10^6^ cells were treated with DMEM containing 1% formaldehyde for 10 min at room temperature for crosslinking. Washing, sonication and immunoprecipitation were performed as described previously.^11^ The antibodies used were directed against H3K9/14Ac (SCB; SC-8655), anti-HDAC1/2 (SCB; SC-7872), TLX (LifeSpan Biosciences; LS-B4564), RNA Pol-II (Diagenode, Seraing, Belgium; C15200004), anti-H3K9me3 (Abcam; ab8898) or mouse/rabbit IgG. Quantitative PCRs (qPCR) were performed using the SYBR Green IQ supermix (Bio-Rad, Hercules, CA, USA) and the ICycler IQ Real-Time Thermal Cycler (Bio-Rad). Percentage of input is calculated and represented from three different experiments.

Primers used are as follows: hMMP-2 – sense, (a) 5′-CACCTCTTTAGCTCTTCA-3′, (b) 5′-TCTCCGGTGTACCTAAGAAC-3′, (c) 5′-AGTACCGCTGCTCTCTAACC-3′, (d) 5′-CAAGGGAGGGCAGCCGCCAGAT-3′; hOCT-4 – sense, (a) 5′-CAGCCACTTAGGAGGCTGGAG-3′, (b) 5′-CGAAGGATGTTTGCCTAATG-3′; actin – sense, 5′-AGTGCAGTGGCGCGATCTCGG-3′, antisense, 5′-TGGCTCACGTCTGTAATC-3′.

The binding of TLX to the MMP-2 promoter was examined with the Universal EZ-TFA Transcription Factor Assay Kit (70-501; Upstate, Millipore, Darmstadt, Germany) according to the vendor's manual. Briefly, 2 pM of 5′-end biotin-labeled consensus oligonucleotide (5′-TAGCTCTTCAGGTCTCAGCTCAG**AAGTCAC**TTCTTCCAGGAAGCCTTCCT-3′ bold letters are putative TLX-binding site) and its reverse from MMP-2 promoter were annealed and used to capture TLX from 12.5 *μ*g of nuclear lysate from IMR-32 cells. A nonspecific capture oligo served as background control, and mouse/rabbit IgG served as background control. Further, two mutant oligos with only the consensus modified (consensus: AAGTCA, Mut1: GGGTCA or Mut2: ACATCA) were used to confirm the specificity of capture. The values obtained are means of three independent experiments along with S.D. as error bars.

### Statistics

Statistical analysis was performed using Student's *t-*test and the Pearson's product–moment correlation coefficient. All data are expressed as mean±S.D. *P*<0.05 was considered statistically significant (**P<0.005 and *P<0.05). All calculations were performed using SigmaPlot (San Jose, CA, USA).

## Abbreviations

ABCG2: ATP binding cassette-G2
bFGF: basic fibroblast growth factor
ChIP: chromatin immunoprecipitation
EGF: epidermal growth factor
EMT: epithelial-to-mesenchymal transition
GAPDH: glyceraldehyde-3-phosphate dehydrogenase
HIF: hypoxia-inducing factor
MMP: matrix metalloproteinase
NB: neuroblastoma
NOD/SID: non-obese diabetic/severe-combined immunodeficiency
PNS: peripheral nervous system
Pol-II: RNA polymerase-II
PTEN: phosphatase and tensin homolog
TIC: tumor-initiating cell
TLX: *Drosophila**tailless* homolog
VEGF: vascular endothelial growth factor
WT: wild type
