# Supplementary material for: TLX activates MMP-2, promotes self-renewal of tumor spheres in neuroblastoma and correlates with poor patient survival
Source: Cell Death Dis. 2014 Oct 30;5(10):e1502–. doi: 10.1038/cddis.2014.449 (PMC4237266; doi:10.1038/cddis.2014.449)
Supplement: Supplementary Information [file cddis2014449x1.doc]

| |  | | --- | |
| --- | --- |

Supplementary Figure 1.


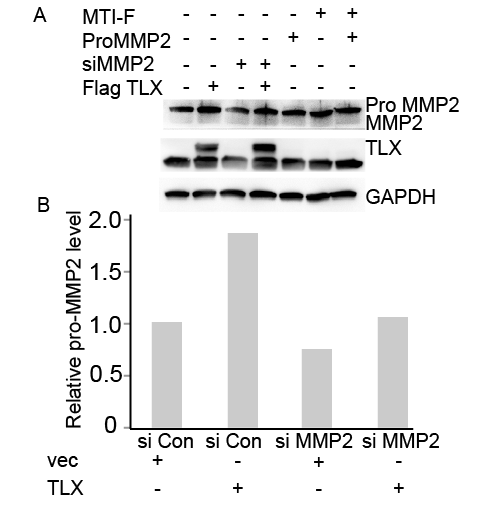


**TLX rescues pro-MMP2 levels in SKN-BE2c cells.**

A. Immunoblot analysis of Pro-MMP2 in SKNBE2 cells with indicated plasmids. B. Graph representing the normalised levels of Pro-MMP2 after rescue by TLX.

**Supplementary Method:** SKNBE2c cells were seeded 300 000 cells per well of 6-well plate and the cells were harvested 48 hours after transfection. Cells were cotransfected with MMP-2 siRNA (Santa Cruz Biotechonologies, sc-29398) and FLAG-tagged TLX using FuGENE HD (Roche). control siRNA FLAG plasmid were used as control vectors. pSG-MT1-F, pSG-MMP2 or both were transfected for positive controls of pro-MMP-2 and MMP-2. Plasmids pSG-MMP2 and pSG-MTI-F were provided by Motoharu Seiki (Division of Cancer Cell Research, Institute of Medical Science, The University of Tokyo, Tokyo, 108-8639, Japan)
