# Supplementary figures and images for: TLX activates MMP-2, promotes self-renewal of tumor spheres in neuroblastoma and correlates with poor patient survival
Source: Cell Death Dis. 2014 Oct 30;5(10):e1502–. doi: 10.1038/cddis.2014.449 (PMC4237266; doi:10.1038/cddis.2014.449)

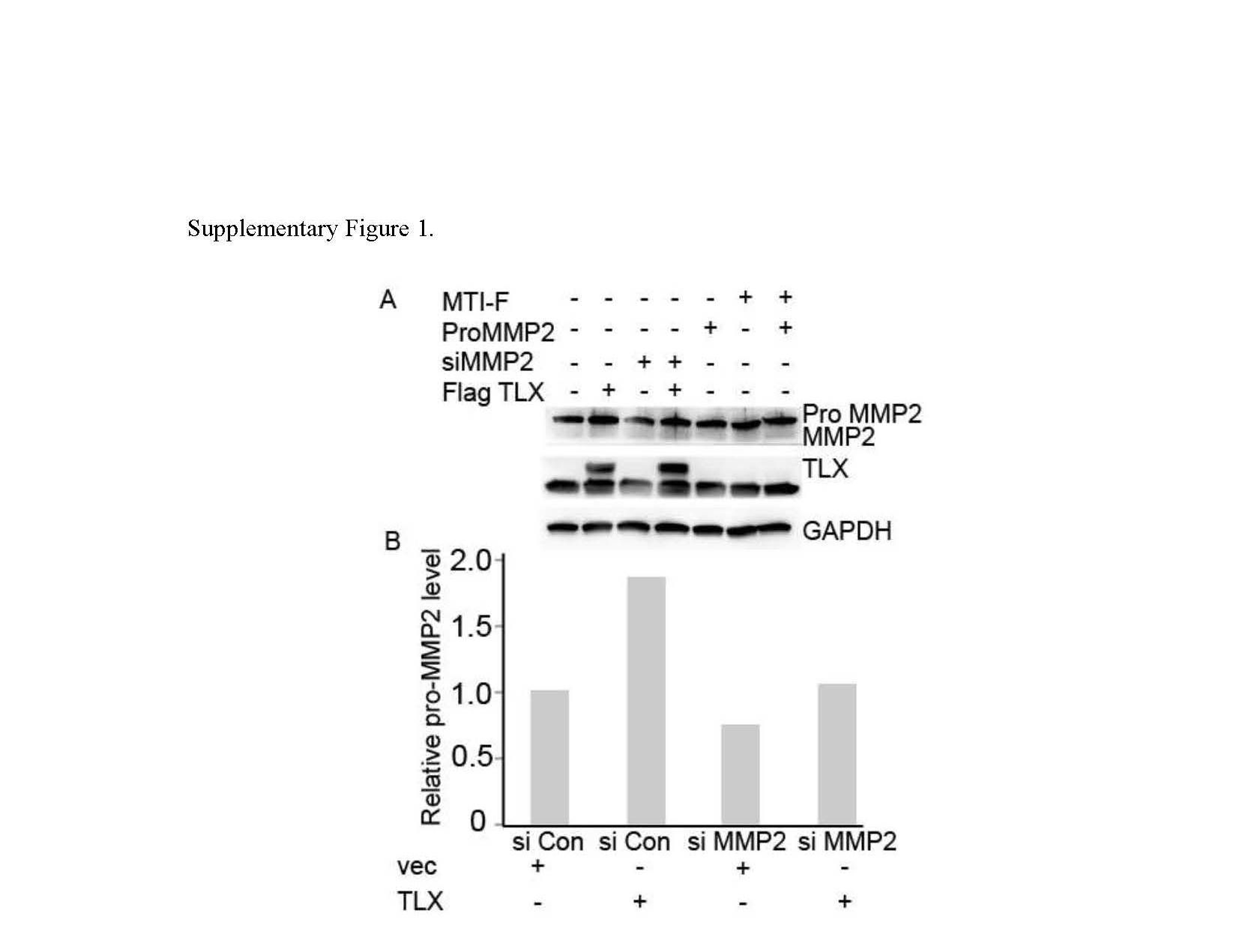

Supplement: Supplementary Figure 1 [file cddis2014449x2.png]
